# 7*S*,15*R*-Dihydroxy-16*S*,17*S*-epoxy-docosapentaenoic Acid Overcomes Chemoresistance of 5-Fluorouracil by Suppressing the Infiltration of Tumor-Associated Macrophages and Inhibiting the Activation of Cancer Stem Cells in a Colorectal Cancer Xenograft Model

**DOI:** 10.3390/md21020080

**Published:** 2023-01-24

**Authors:** Yan Su, Hack Sun Choi, Jong Hyun Choi, Hee-Sik Kim, Yong-Suk Jang, Jeong-Woo Seo

**Affiliations:** 1 Microbial Biotechnology Research Center, Korea Research Institute of Bioscience and Biotechnology (KRIBB), Jeongeup-si 56212, Republic of Korea; 2 Department of Bioactive Material Sciences, The Institute for Molecular Biology and Genetics, Jeonbuk National University, Jeonju-si 54896, Republic of Korea; 3 Faculty of Biotechnology, College of Applied Life Sciences, Jeju National University, Jeju-si 63243, Republic of Korea; 4 Cell Factory Research Center, Korea Research Institute of Bioscience and Biotechnology (KRIBB), Daejeon-si 34141, Republic of Korea

**Keywords:** colorectal cancer, 5-fluorouracil, 7*S*,15*R*-dihydroxy-16*S*,17*S*-epoxy-docosapentaenoic acid, tumor-associated macrophages, chemoresistance

## Abstract

Although the tumor bulk is initially reduced by 5-fluorouracil (5-FU), chemoresistance developed due to prolonged chemotherapy in colorectal cancer (CRC). The enrichment of cancer stem cells (CSCs) and the infiltration of tumor-associated macrophages (TAMs) contribute to chemoresistance and poor outcomes. A docosahexaenoic acid derivative developed by our group, 7*S*,15*R*-dihydroxy-16*S*,17*S*-epoxy-docosapentaenoic acid (diHEP-DPA), exerts antitumor effects against TAMs infiltration and CSCs enrichment in our previous study. The current study aimed to investigate whether diHEP-DPA was able to overcome chemoresistance to 5-FU in CRCs, together with the potential synergistic mechanisms in a CT26-BALB/c mouse model. Our results suggested that although 5-FU inhibited tumor growth, 5-FU enriched CSCs via the WNT/β-catenin signaling pathway, resulting in chemoresistance in CRCs. However, we revealed that 5-FU promoted the infiltration of TAMs via the NF-kB signaling pathway and improved epithelial–mesenchymal transition (EMT) via the signal transducer and activator of the transcription 3 (STAT3) signaling pathway; these traits were believed to contribute to CSC activation. Furthermore, supplementation with diHEP-DPA could overcome drug resistance by decreasing the CSCs, suppressing the infiltration of TAMs, and inhibiting EMT progression. Additionally, the combinatorial treatment of diHEP-DPA and 5-FU effectively enhanced phagocytosis by blocking the CD47/signal regulatory protein alpha (SIRPα) axis. These findings present that diHEP-DPA is a potential therapeutic supplement to improve drug outcomes and suppress chemoresistance associated with the current 5-FU-based therapies for colorectal cancer.

## 1. Introduction

Colorectal cancer (CRC) is a highly malignant cancer that, with the aging of the population and changes in lifestyle, accounts for a third of the cancer-related deaths worldwide [1]. One of the most important first-line medicines in clinical CRC therapy is 5- fluorouracil (5-FU). However, chemoresistance and the associated adverse effects have limited the clinical benefits; thus, it is imperative to find alternative therapies to overcome chemoresistance and improve outcomes with less toxicity for patients with CRC [2]. 

Several studies have reported that long-term treatment with 5-FU may cause a cytokine storm in the tumor microenvironment (TME), induce macrophages to produce proinflammatory mediators, and enrich cancer stem cells (CSCs), resulting in poor outcomes and chemoresistance [3,4,5]. Therefore, the biological factors involved in mediating resistance to 5-FU-based therapy must be extensively examined.

The TME, which comprises tumor cells and stromal cells, including fibroblasts, endothelial cells, and immune cells, plays a crucial role in tumor progression, including metastasis, invasion, stemness, and acquired chemoresistance [6,7]. Tumor-associated macrophages (TAMs) are major components of the TME. Macrophages are classified as M1 and M2 subtypes, depending on their immune responses [8]. M2-like macrophages are the main subsets of TAMs, and they produce various growth factors and cytokines, such as vascular endothelial growth factor (VEGF), interleukin-6 (IL-6), and tumor necrosis factor-α (TNF-α). These not only promote tumor growth but also confer chemoresistance [9,10].

During the epithelial–mesenchymal transition (EMT) procession, epithelial cancer cells acquire the features of mesenchymal cells by downregulating epithelial markers (E-cadherin) and upregulating mesenchymal markers (vimentin and N-cadherin) [11]. Cancer cells undergoing EMT reportedly possess enhanced metastatic and invasive abilities, express high levels of stem surface markers, and strongly resist radio- or chemotherapy [12]. Several cytokines, chemokines, and growth factors secreted by TAMs likely contribute to EMT and chemoresistance by activating the signal transducer and activator of the transcription 3 (STAT3) pathway in many tumors [13,14].

CD47 is a common mechanism through which cells protect themselves from phagocytosis. CD47 functions as a ligand for signal regulatory protein-α (SIRPα), a protein expressed on macrophages and dendritic cells. SIRPα binds with CD47 to initiate a signaling cascade which results in the inhibition of phagocytosis, a critical “do not eat me” signal for the innate immune system which is overexpressed on many kinds of tumor cells [15,16].

CSCs, a subpopulation of cells, are characterized by some key markers, such as CD133, CD166, CD44, CD24, SOX2, OCT4, and ALDH, which are associated with tumor initiation, growth, and therapy resistance [17]. Some studies also showed that massive populations of CSCs correlate with poor overall survival rates after chemotherapy in advanced patients with CRC [18]. One of the mechanisms of activation of CSCs involves the WNT/β-catenin pathway [17]. Treatment using 5-FU activated CSCs via p53-induced WNT3 transcription, followed by the activation of the WNT/β-catenin pathway in CRC cell lines and xenograft tumors as well as patient avatar models [19].

**Figure 1:**
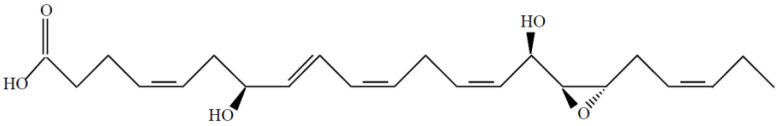
The structure of 7*S*,15*R*-dihydroxy-16*S*,17*S*-epoxy-docosapentaenoic acid (diHEP-DPA).

The novel resolvin, 7S,15R-dihydroxy-16S,17S-epoxy-docosapentaenoic acid (diHEP-DPA) (Figure 1), is synthesized by cyanobacterial lipoxygenase from DHA (purity > 98%). In our previous study, we showed the generation and structure of diHEP-DPA and demonstrated that diHEP-DPA regulated the polarization of TAMs, EMT, and CSCs activation in HCT116 and HT26 cells [20]. In the current study, we further investigated the effects of diHEP-DPA on 5-FU-induced chemoresistance in colorectal xenograft tumors via TAMs, CSCs, and EMT. Our results collectively indicated that diHEP-DPA reversed the challenges caused by prolonged chemotherapy, such as the infiltration of M2-like TAMs, the enrichment of CSCs, and the progression of EMT in colorectal tumors, suggesting that diHEP-DPA is a promising therapy to overcome 5-FU resistance and improve 5-FU treatment outcomes in colon cancer.

## 2. Results

### 2.1. diHEP-DPA and 5-FU Inhibited Tumor Growth in the CRC Xenograft Model

**Figure 2:**
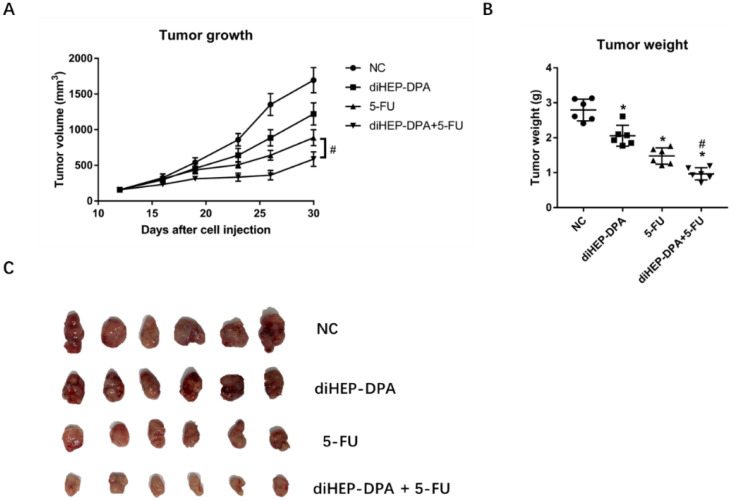
Effects of diHEP-DPA and 5-FU on tumor growth and tumor weight. (**A**) Tumor mass volumes in BALB/c mice intraperitoneally injected with saline (control), diHEP-DPA (10 μg/kg/day), 5-FU (20 mg/kg/five times a week), or both diHEP-DPA (10 μg/kg/day) and 5-FU (20 mg/kg/five times a week). (**B**) Tumor weight after sacrifice on day 30. (**C**) Tumors excised after sacrifice on day 30. Data are expressed as mean ± SD; *n* = 6 per group. * *p* < 0.05 compared with the control group; ^#^ *p* < 0.05 compared with the 5-FU group.

The combined treatment of diHEP-DPA and 5-FU synergistically inhibited tumor growth and size (Figure 2). Briefly, the tumor volume in control mice was ~1791 mm^3^ (2.80 ± 0.30 g), as measured on day 30 post-cell injection. The corresponding volumes in the diHEP-DPA, 5-FU, and combined treatment groups were ~1221 mm^3^ (2.07 ± 0.34 g), ~887 mm^3^ (1.48 ± 0.23 g), and ~636 mm^3^ (0.96 ± 0.13 g). These findings indicated that, compared with diHEP-DPA or 5-FU alone, the combination of the two inhibited colorectal tumor growth and size more effectively.

### 2.2. diHEP-DPA Suppressed 5-FU-Induced CSCs Activation via the WNT/β-Catenin Pathway

**Figure 3:**
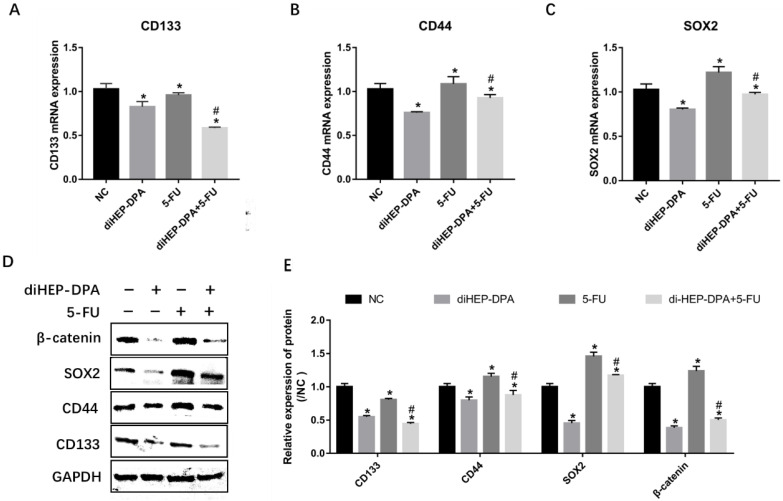
Effects of diHEP-DPA and 5-FU on cancer stem cells via the WNT/β-catenin signaling pathway. Relative mRNA levels of (**A**) CD133, (**B**) CD44, and (**C**) SOX2 were determined by qRT-PCR in isolated tumors. (**D**) The protein levels of CD133, CD44, SOX2, and β-catenin were detected by western blotting in isolated tumors. (**E**) The relative levels of CD133, CD44, SOX2, and β-catenin were calculated by ImageJ. Data are expressed as mean ± SD; *n* = 3 per group. * *p* < 0.05 compared with the control group; # *p* < 0.05 compared with the 5-FU group.

As shown in Figure 3A–C, diHEP-DPA significantly decreased the expression of CSC markers, including CD133, CD44, and SOX2. However, the expression of CD133 was significantly inhibited, whereas the expressions of CD44 and SOX2 were markedly increased in the 5-FU group. Combination treatment with diHEP-DPA and 5-FU reversed the 5-FU-induced activation of CSC markers. To confirm the above results, these protein levels were evaluated via western blotting. Similarly, diHEP-DPA significantly decreased the protein levels of all CSC markers. Although the 5-FU treatment decreased the expression of CD133, it significantly increased the protein expressions of CD44 and SOX2. Moreover, the combined treatment decreased the levels of these CSC markers more effectively than the 5-FU treatment (Figure 3D). We assumed that 5-FU enriched CSCs by the activation of the WNT/β-catenin pathway. diHEP-DPA caused a significant decrease in the β-catenin level. However, the 5-FU treatment significantly increased the β-catenin level, which was reversed via the additional diHEP-DPA treatment (Figure 3D). It predominantly suggested that diHEP-DPA overcame 5-FU-induced CRC activation via the WNT/β-catenin signaling pathway.

### 2.3. diHEP-DPA Inhibited 5-FU-Induced Infiltration of M2-likeTAMs 

**Figure 4:**
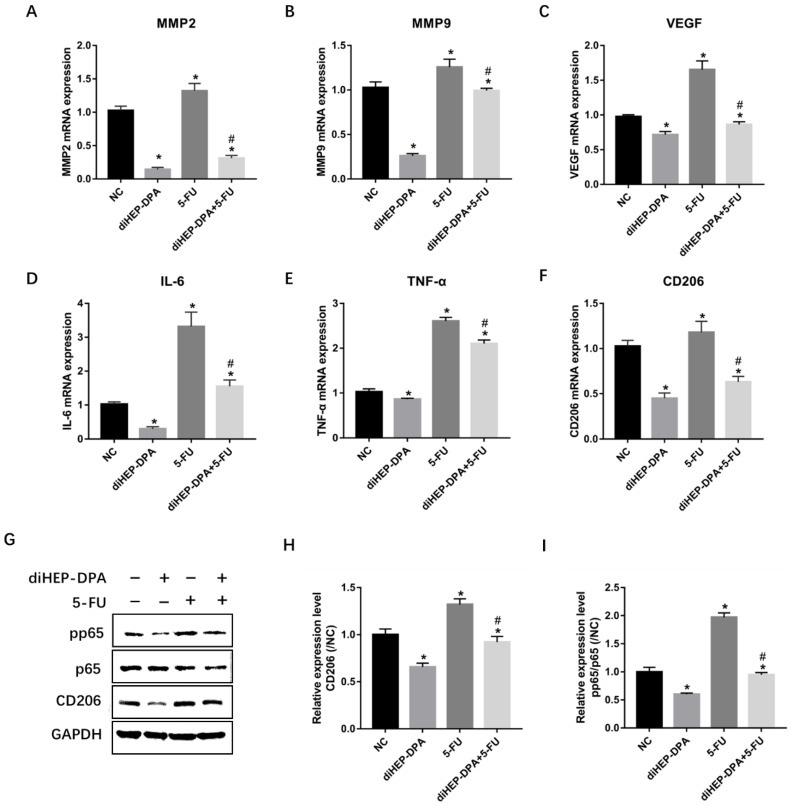
Effects of diHEP-DPA and 5-FU on the infiltration of M2-like TAMs and the NF-κB signaling pathway. Relative mRNA levels of TAMs secretions including (**A**) MMP2, (**B**) MMP9, (**C**) VEGF, (**D**) IL-6, and (**E**) TNF-α in isolated tumors. Relative mRNA levels of (**F**) CD206 were determined via qRT-PCR. (**G**) The protein levels of CD206, p65, and pp65 were detected by western blotting in isolated tumors. The relative levels of (**H**) CD206 and (**I**) pp65/p65 were calculated using ImageJ. Data are expressed as mean ± SD; *n* = 3 per group. * *p* < 0.05 compared with the control group; ^#^ *p* < 0.05 compared with the 5-FU group.

The crucial proteolytic enzymes, growth factors, and inflammatory cytokines secreted by TAMs, including MMP2, MMP9, VEGF, IL-6, and TNF-α, were significantly downregulated by diHEPA-DPA but upregulated by the 5-FU treatment. The combination treatment, again, significantly lowered the expression of these factors compared to the 5-FU treatment (Figure 4A–E). Furthermore, the treatment with diHEP-DPA significantly downregulated the expression of CD206, a specific marker of M2-likeTAMs, indicating that it could suppress the infiltration of M2-like TAMs into tumor tissue. In contrast, the 5-FU treatment significantly increased CD206 levels, which were decreased by the combination treatment (Figure 4F,G,H). The NF-κB signaling pathway, which includes NF-kB1 (p50), NF-kB2 (p52), RelA (p65), RelB, and c-Rel, plays a crucial role in immune responses, cellular growth, apoptosis, and inflammation [20]. The phosphorylation of NF-kB (pp65) was significantly reduced in diHEP-DPA, but elevated in 5-FU-treated tumors, and subsequently reduced after the additional diHEP-DPA treatment (Figure 4G,I). All these findings suggested that diHEP-DPA inhibited the 5-FU-induced infiltration of M2-like TAMs and the NF-κB signaling pathway.

### 2.4. diHEP-DPA Impeded EMT via the STAT3 Signaling Pathway

**Figure 5:**
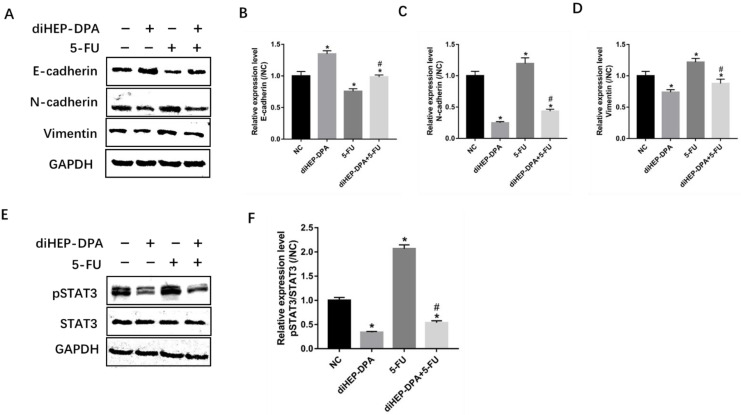
Effects of diHEP-DPA and 5-FU on EMT via the STAT3 signaling pathway. (**A**) The level of E-cadherin, N-cadherin, and vimentin were detected by western blotting. The relative expression of (**B**) E-cadherin (**C**) N-cadherin, and (**D**) vimentin were calculated by ImageJ. (**E**) The level of STAT3 and pSTAT3 were detected by western blotting in isolated tumors and (**F**) were calculated by ImageJ. Data are expressed as mean ± SD; *n* = 3 per group. * *p* < 0.05 compared with the control group; ^#^ *p* < 0.05 compared with the 5-FU group.

EMT is closely related to increased chemoresistance and tumor metastasis [21]. As shown in Figure 5A–D, diHEP-DPA upregulated the expression of the epithelial marker E-cadherin, and downregulated mesenchymal markers N-cadherin and vimentin, while 5-FU treatment enhanced the expression of N-cadherin and vimentin and declined the expressions of E-cadherin. Furthermore, we observed a significantly decreased expression of N-cadherin and vimentin and an increased expression of E-cadherin in the combination treatment group compared with the 5-FU group.

The STAT3 signaling pathway has been shown to stimulate EMT progression [22,23]. To assess the mechanism by which this stimulation occurs, we examined the expression of pSTAT3 via western blotting. We observed that diHEP-DPA alone could significantly downregulate the expression of pSTAT3, while the 5-FU treatment significantly enhanced it. The combination treatment decreased pSTAT3 levels compared with the 5-FU treatment (Figure 5E,F), showing that diHEP-DPA suppressed 5-FU-induced EMT progression by inhibiting the STAT3 signaling pathway.

### 2.5. diHEP-DPA and 5-FU Enhanced Macrophage Phagocytic Activity via CD47/SIRPα

**Figure 6:**
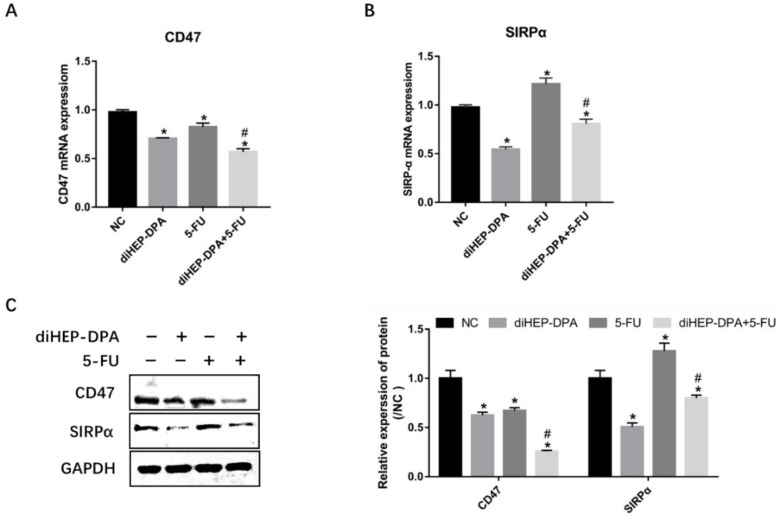
Effects of diHEP-DPA and 5-FU on phagocytic activity via the CD47/SIRPα axis. Relative mRNA levels of (**A**) CD47 and (**B**) SIRPα were determined by qRT-PCR in isolated tumors. (**C**) The protein levels of CD47 and SIRPα were detected by western blotting and calculated by ImageJ in isolated tumors. Data are expressed as mean ± SD; *n* = 3 per group. * *p* < 0.05 compared with the control group; ^#^ *p* < 0.05 compared with the 5-FU group.

The disruption of the CD47/SIRPα axis reduces the ability of the tumor to escape phagocytosis [24]. Hence, we evaluated the effects of diHEP-DPA and 5-FU on SIRPα and CD47 expression in tumor tissues. diHEP-DPA alone significantly reduced the gene levels of CD47 and SIRPα compared with the control; 5-FU downregulated the expression of CD47 but upregulated the expression of SIRPα; the combination of diHEP-DPA and 5-FU was proven to downregulate the expression of CD47 and SIRPα compared with the 5-FU group (Figure 6A,B). Accordingly, the western blotting results showed similar trends (Figure 6C). These findings demonstrated the powerful effect of diHEP-DPA combined with the 5-FU on phagocytic activity via the CD47/SIRPα axis.

## 3. Discussion

Since the 1950s, 5-FU has been an antitumor drug widely used to treat different types of cancer, including colorectal cancer and breast cancer [2]. However, because post-chemotherapy recurrence reduces clinical outcomes even for those who initially react to chemotherapy, there is an urgent need to develop new medications [19].

Cancer progression depends on many different molecular pathways, which hints at the complexity of the complex mechanisms of treatment resistance [25]. Additionally, most drug resistance studies have focused on the genetics and epigenetics of cancer cells, while ignoring the cellular components of the TME, among which macrophages are the most prominent innate immune cells [26]. Our present study uncovered important mechanisms of resistance to 5-FU therapy related to CSCs and TAMs. Firstly, we found that the resistance of 5-FU therapy involves the enrichment of CSCs related to the activation of WNT/β-catenin signaling in the CT-26-BALB/c model. Moreover, we identified that 5-FU induced the infiltration of M2-like macrophages and promoted the EMT progression, which contribute to the resistance of 5-FU in CRC. Importantly, diHEP-DPA could overcome the chemoresistance caused by 5-FU via depleting CSCs, inhibiting the infiltration of M2-like TAMs, and suppressing the EMT progression. Moreover, combinatorial treatment with diHEP-DPA and 5-FU enhanced macrophage phagocytosis and effectively repressed tumor regrowth. 

Although the 5-FU treatment could effectively inhibit colorectal tumor growth, we observed an enrichment of CSCs in 5-FU-treated tumors supported by the upregulation of CSC markers (CD44, SOX2), which could be responsible for the observed chemoresistance. Interestingly, although the 5-FU treatment downregulated the expression of CD133, it is reported that CD133 expression is not restricted to cancer-initiating cells; further, during the metastatic transition, CD133^+^ tumor cells may give rise to the more aggressive CD133^–^ subset (such as CD44+/CD24−/CD133−), taking away the necessity for CD133 in tumor initiation [27]. The level of SOX2 expression in CRC is believed to confer to tumor metastasis and lymph node infiltration [28]. The WNT/β-catenin signaling pathway activates CSCs, contributing to tumor initiation and tumor recurrence [19,28]. Indeed, we found that the 5-FU treatment upregulated the expression of β-catenin, the center of the WNT/β-catenin signaling pathway, further activating CSCs. Similarly, another previous study demonstrated that 5-FU activated CSCs via the p53-mediated WNT/β-catenin pathway in CRC cell lines, xenograft tumors, and patient avatar models [19]. Interestingly, diHEP-DPA could reverse the upregulation of β-catenin expression caused by 5-FU and then reduce the enrichment of CSCs, minimizing recurrence as an expansion of CSCs. The tankyrase inhibitor XAV939 reversed 5-fluorouracil chemoresistance by targeting the WNT/β-catenin signaling pathway in colorectal cancer cells [29]. Our results have provided an experimental basis for the clinical application of diHEP-DPA in combination with 5-Fu-based chemotherapeutics for CRC patients, especially for those with a poor chemotherapy tolerance. 

TAMs are another key factor for chemoresistance, which has been previously recognized by many researchers; TAMs release several enzymes, cytokines, chemokines, and growth factors which contribute to tumor growth and chemoresistance, making them potential targets to inhibit tumor recurrence [8,30,31]. Recently, it has been proposed that CSCs can directly or indirectly interact with several immune cell populations within the tumor microenvironment, which are thought to markedly influence tumor progression [32,33]. Therefore, strategies aimed at depleting TAMs carry the promise of increasing chemotherapy efficiency and decreasing anti-cancer drug resistance [3,9,34]. In this study, we found that diHEP-DPA significantly inhibited the infiltration of TAMs and decreased the secretion of MMP2, MMP9, VEGF, IL-6, and TNF-α in tumor tissue. Nevertheless, the 5-FU treatment increased the infiltration of TAMs and secreted higher levels of these factors, which contributed to the upregulation of CSCs. Similarly, the 5-FU treatment significantly increased the infiltration of TAMs, and produced ornithine decarboxylase-dependent putrescine to confer resistance to further chemotherapy with 5-FU in CRC [7]. The 5-FU treatment induced the activation of NF-κB and upregulated CSCs in HCT116 high-density tumor microenvironment co-cultures, which were abolished by curcumin [34]. NF-κB is overactivated in tumors and controls tumor survival, metastasis, and chemoresistance. It has been proven that blocking the NF-κB signaling pathway could restore the sensitivity towards 5-FU in CRC [35,36]. We observed that 5-FU promoted the phosphorylation of p65, which may be activated by proinflammatory cytokines secreted by TAMs, such as IL-6 and TNFα, which were then abolished by diHEP-DPA. Based on these results, we hypothesized that the aberrant activation of NF-κB induced by 5-FU was one of the major causes leading to CRC chemoresistance and that diHEPA-DPA possibly abolished the 5-FU-induced abnormal activation of NF-κB. Similarly, aspirin enhanced the sensitivity to 5-FU in CRC by suppressing the NF-κB pathway both in vivo and in vitro [37]. The combination of curcumin and 5-FU has synergistic anti-tumor or modulatory effects on HCT116 and their 5-FU-chemoresistant counterparts via blocking the activity of NF-κB [38]. These results indicate that the interaction between the tumor and TAMs is crucial in promoting CSCs and that there is a strong chemotherapy sensitivity of diHEP-DPA by blocking NF-κB and suppressing TAMs infiltration. 

Cytokines secreted by TAMs also stimulate EMT progression, which contributes to tumor invasion, metastasis, and chemoresistance [3,21,39]. We evaluated the effect of diHEP-DPA, 5-FU, and the combined use of both on the EMT process. Interestingly, we found that diHEP-DPA suppressed EMT by upregulating the epithelial cell marker E-cadherin and downregulating the mesenchymal cell markers (vimentin and N-cadherin), which is consistent with the findings of our previous in vitro study [20]. Moreover, it is reported that M2-like macrophages regulated 5-FU resistance in CRC cells through the epithelial–mesenchymal transition (EMT) program [40]. In current study, we observed that 5-FU increased the infiltration of M2-like TAMs in tumor tissue; thus, we assumed that 5-FU treatment might promote the EMT process. We revealed that 5-FU strongly decreased the expression of E-cadherin and increased the expression of vimentin and N-cadherin in the colorectal tumor tissue, indicating a promotion of the EMT status. Importantly, diHEP-DPA could significantly inhibit 5-FU-induced EMT progression. Curcumin enhanced the inhibitory effects of 5-FU against the cell growth on 5-FU resistant HCT-116 cells by regulating the TET1-NKD-Wnt signal pathway to inhibit the EMT progress [41]. Enalapril has been shown to enhance the antitumor efficacy of 5-FU and counter chemoresistance in CRC by suppressing cell proliferation, EMT, and chemoresistance via the NF-κB/STAT3 pathway in cells and nude mice [22]. As expected, we observed the overexpression of pp65 and pSTAT3 in 5-FU-treated tumors. Combined treatment with diHEP-DPA effectively inhibited the activation of NF-κB and STAT3, subsequently enhancing the effects of chemotherapy and preventing chemoresistance in CRC. These results strongly support that diHEP-DPA blocks the STAT3 signaling pathway, thus inhibiting the EMT progression indued by 5-FU.

The CD47/SIRPα interaction is another therapeutic target for human solid tumors: CD47 is a unique cell-surface marker expressed by human cancers; SIRPα is a protein expressed on macrophages and dendritic cells [15,16,24]. In this study, we demonstrated that diHEP-DPA inhibited the expression of CD47 and SIRPα. The expression of CD47 was only decreased by 5-FU; however, it increased the expression of SIRPα. The upregulation of SIRPα might be correlated with the infiltration of TAMs; in turn, high levels of SIRPα promote TAMs polarization [42]. However, the combined treatment of diHEP-DPA and 5-FU resulted in a synergistic suppression of the CD47/SIRPα axis. Our data clearly suggests the synergistic increase of phagocytosis between diHEP-DPA and 5-FU by blocking the CD47/ SIRPα axis.

## 4. Materials and Methods

### 4.1. Materials

The mouse colon cancer cell line CT26 was purchased from the Korean Cell Line Bank (Seoul, Korea). The bicinchoninic acid assay kit was purchased from Sango Company (San Diego, CA, USA). Antibodies were purchased from Abcam (Cambridge, MA, USA). The enhanced chemiluminescence (ECL) substrate kit was purchased from Bio-Rad Laboratories Inc. (Tewksbury, MA, USA).

### 4.2. Animals and Cell Culture

Five-week-old female mice obtained from Orient bio (Gyeonggi, Korea) were housed at a constant temperature (21–23 °C) and a relative humidity (60–70%) under a 12 h light/dark cycle. The study was reviewed by the Institutional Animal Care and the Use Committee of the Korea Research Institute of Bioscience and Biotechnology (Daejeon, Korea), and was approved by the Institutional Animal Ethics Committee (KRIBB-AEC-22140).

CT26 cells were maintained in Roswell Park Memorial Institute 1640 medium, supplemented with 10% fetal bovine serum (HyClone, Thermofisher Scientific, Waltham, MA, USA), 100 U/mL penicillin, and 100 μg/mL streptomycin (Gibco, Thermofisher Scientific), at 37 °C in 5% CO_2_. 

### 4.3. CRC Xenograft Model in BALB/c Mice

Female mice were injected subcutaneously in the right flank with CT26 cells (3 × 10^4^ cells/mouse), suspended in 100 μL of phosphate-buffered saline. When the mean tumor size reached between 100–150 mm^3^, the mice were randomly divided into four groups: (1) control, intraperitoneally administered with sterile saline; (2) diHEP-DPA, intraperitoneally administered 10 μg/kg of diHEP-DPA dissolved in saline daily; (3) 5-FU, intraperitoneally administered 20 mg/kg of 5-FU dissolved in saline, five times a week; (4) diHEP-DPA + 5-FU, intraperitoneally administered 10 μg/kg of diHEP-DPA daily and 20 mg/kg of 5-FU five times a week. The tumor size was calculated twice a week and the mice were sacrificed when the tumor volume without treatment grew to approximately 2000 mm^3^.

### 4.4. Quantitative Reverse Transcription PCR (qRT-PCR)

Total RNA was isolated using a TaKaRa MiniBEST kit (TaKaRa, Tokyo, Japan), according to the manufacturer’s protocol. The levels of transcripts were measured with a One-Step AccuPower GreenStar RT-qPCR PreMix kit (Bioneer Corporation, Daejeon, Korea) using SYBR Green, according to the manufacturer’s instructions. RT-PCR was performed in a reaction volume of 50 μL. qRT-PCR was performed in the CFX Connect system (Bio-Rad, CA, USA). The glyceraldehyde-3-phosphate dehydrogenase (GAPDH) gene was used as a housekeeping control. The relative mRNA expression of target genes was calculated by the 2^−ΔΔCT^ method. All specific primers are listed in Table 1.

### 4.5. PROTEIN Preparation and Western Blotting

The protein was obtained from tumor tissue homogenates using a RIPA lysis buffer (Biosolution, Seoul, Korea) supplemented with 1:100 each of protease inhibitor cocktail, phenylmethyl sulfonyl fluoride, and phosphatase inhibitor. The tissue homogenates were incubated on ice for 30 min and the concentration of the protein was estimated using a bicinchoninic acid assay kit (Abcam, Cambridge, MA, USA). The samples were heated with a loading buffer (Solarbio, Beijing, China) for 10 min at 100 °C. Equal amounts of proteins (~80 μg) were separated on 7.5% or 10% sodium dodecyl sulfate–polyacrylamide gels and transferred onto 0.45 μm polyvinylidene fluoride membranes (Millipore, Bedford, MA, USA) at 20 V for 120 min. The membranes were blocked for 45 min with Tris-buffered saline/Tween 20 (TBST) containing 5% skim milk and incubated overnight at 4 °C with primary antibodies against the following proteins: β-catenin (ab223075, 1:10,000), CD133 (ab284389, 1:5000), CD44 (ab189524, 1:1000), SOX2 (ab92494, 1:1000), CD206 (ab64693, 1:5000), p65 (ab16502, 1:10,000), pp65 (ab76302, 1:1000), STAT3 (ab68153, 1:1000), pSTAT3 (ab76315, 1:2000), E-cadherin (ab231303, 1:1000), N-cadherin (ab76011, 1:5000), vimentin (ab92547, 1:1000), CD47 (ab214453, 1:1000), SIRPα (ab191419, 1:1000), and GAPDH (ab181602, 1:20,000). The membranes were washed thrice with TBST before being incubated at room temperature for 2 h with the appropriate secondary antibodies (ab205718, 1:50,000). After being rinsed with TBST, the membranes were incubated with the Clarity Western ECL Substrate (Bio-Rad, Hercules, CA, USA). The CL-XPosure film was then exposed to the polyvinylidene fluoride membranes (Thermo Scientific, Rockford, IL, USA). 

### 4.6. Statistical Analysis

The data were expressed as means ± standard deviation. Statistical analysis was performed by one-way ANOVA analysis of variance using GraphPad Prism 7.0 (GraphPad, San Diego, CA, USA). The results were considered statistically significant for *p*-values < 0.05.

## 5. Conclusions

This study aimed to evaluate the effects of diHEP-DPA on 5-FU-induced chemoresistance in CRC. Per our findings, diHEP-DPA could be an ideal CRC therapy to strongly restore chemosensitivity and significantly boost the antitumor effect of 5-FU. Our results have provided an experimental basis for the clinical application of diHEP-DPA in combination with 5-FU-based chemotherapeutics for patients with CRC, especially for those with poor chemotherapeutic tolerance. Considering the low toxicity and the better economic effectiveness of the combination therapy, this study is of high clinical value. Further clinical studies are necessary to confirm our findings in patients with CRC to precede a translation of our treatment strategy to clinical oncology. 

## Figures and Tables

**Table 1:** Primer sequences.

Gene (Mouse)	Sequence (5′ → 3′)
CD133	Forward: CTGCGATAGCATCAGACCAAGC
	Reverse: CTTTTGACGAGGCTCTCCAGATC
CD44	Forward: CGGAACCACAGCCTCCTTTCAA
	Reverse: TGCCATCCGTTCTGAAACCACG
SOX2	Forward: AACGGCAGCTACAGCATGATGC
	Reverse: CGAGCTGGTCATGGAGTTGTAC
CD206	Forward: GTTCACCTGGAGTGATGGTTCTC
	Reverse: AGGACATGCCAGGGTCACCTTT
VEGF	Forward: CTGCTGTAACGATGAAGCCCTG
	Reverse: GCTGTAGGAAGCTCATCTCTCC
MMP2	Forward: CAAGGATGGACTCCTGGCACAT
	Reverse: TACTCGCCATCAGCGTTCCCAT
MMP9	Forward: GCTGACTACGATAAGGACGGCA
	Reverse: TAGTGGTGCAGGCAGAGTAGGA
IL-6	Forward: TACCACTTCACAAGTCGGAGGC
	Reverse: CTGCAAGTGCATCATCGTTGTTC
TNF-α	Forward: GGTGCCTATGTCTCAGCCTCTT
	Reverse: GCCATAGAACTGATGAGAGGGAG
CD47	Forward: GGTGGGAAACTACACTTGCGAAG
	Reverse: CTCCTCGTAAGAACAGGCTGATC
SIRPα	Forward: TCATCTGCGAGGTAGCCCACAT
	Reverse: ACTGTTGGGTGACCTTCACGGT
GAPDH	Forward: CATCACTGCCACCCAGAAGACTG
	Reverse: ATGCCAGTGAGCTTCCCGTTCAG

CD, cluster of differentiation; SOX2, SRY-Box transcription factor 2; VEGF, vascular endothelial growth factor; MMP, matrix metalloproteinase; IL-6, interleukin-6; TNF-α, tumor necrosis factor-α; SIRPα, signal regulatory protein alpha; GAPDH, glyceraldehyde-3-phosphate hydrogenase.

## Data Availability

The data presented in this study are available on request from the corresponding author.

## References

[1] Siegel R.L., Miller K.D., Jemal A. (2019). Cancer Statistics, 2019. CA Cancer J. Clin..

[2] Blondy S., David V., Verdier M., Mathonnet M., Perraud A., Christou N. (2020). 5-Fluorouracil Resistance Mechanisms in Colorectal Cancer: From Classical Pathways to Promising Processes. Cancer Sci..

[3] Dean M., Fojo T., Bates S. (2005). Tumour Stem Cells and Drug Resistance. Nat. Rev. Cancer.

[4] Erin N., Grahovac J., Brozovic A., Efferth T. (2020). Tumor Microenvironment and Epithelial Mesenchymal Transition as Targets to Overcome Tumor Multidrug Resistance. Drug Resist. Updates.

[5] Sethy C., Kundu C.N. (2021). 5-Fluorouracil (5-FU) Resistance and the New Strategy to Enhance the Sensitivity against Cancer: Implication of DNA Repair Inhibition. Biomed. Pharmacother..

[6] Acharyya S., Oskarsson T., Vanharanta S., Malladi S., Kim J., Morris P.G., Manova-Todorova K., Leversha M., Hogg N., Seshan V.E. (2012). A CXCL1 Paracrine Network Links Cancer Chemoresistance and Metastasis. Cell.

[7] Zhang X., Chen Y., Hao L., Hou A., Chen X., Li Y., Wang R., Luo P., Ruan Z., Ou J. (2016). Macrophages Induce Resistance to 5-Fluorouracil Chemotherapy in Colorectal Cancer through the Release of Putrescine. Cancer Lett..

[8] Azwar S., Seow H.F., Abdullah M., Jabar M.F., Mohtarrudin N. (2021). Recent Updates on Mechanisms of Resistance to 5-Fluorouracil and Reversal Strategies in Colon Cancer Treatment. Biology.

[9] Shih J.-Y., Yuan A., Chen J.J.-W., Yang P.-C. (2006). Tumor-Associated Macrophage: Its Role in Cancer Invasion and Metastasis. J. Cancer Mol..

[10] Ding L., Liang G., Yao Z., Zhang J., Liu R., Chen H., Zhou Y., Wu H., Yang B., He Q. (2015). Metformin Prevents Cancer Metastasis by Inhibiting M2-like Polarization of Tumor Associated Macrophages. Oncotarget.

[11] Wei H., Liang F., Cheng W., Zhou R., Wu X., Feng Y., Wang Y. (2017). The Mechanisms for Lung Cancer Risk of PM2.5: Induction of Epithelial-Mesenchymal Transition and Cancer Stem Cell Properties in Human Non-Small Cell Lung Cancer Cells. Environ. Toxicol..

[12] Wang W., Zhao Y., Yao S., Cui X., Pan W., Huang W., Gao J., Dong T., Zhang S. (2017). Nigericin Inhibits Epithelial Ovarian Cancer Metastasis by Suppressing the Cell Cycle and Epithelial−mesenchymal Transition. Biochem. Mosc..

[13] Kong L., Zhou Y., Bu H., Lv T., Shi Y., Yang J. (2016). Deletion of Interleukin-6 in Monocytes/Macrophages Suppresses the Initiation of Hepatocellular Carcinoma in Mice. J. Exp. Clin. Cancer Res..

[14] Storr S.J., Safuan S., Ahmad N., El-Refaee M., Jackson A.M., Martin S.G. (2017). Macrophage-Derived Interleukin-1beta Promotes Human Breast Cancer Cell Migration and Lymphatic Adhesion in Vitro. Cancer Immunol. Immunother..

[15] Willingham S.B., Volkmer J.-P., Gentles A.J., Sahoo D., Dalerba P., Mitra S.S., Wang J., Contreras-Trujillo H., Martin R., Cohen J.D. (2012). The CD47-Signal Regulatory Protein Alpha (SIRPa) Interaction Is a Therapeutic Target for Human Solid Tumors. Proc. Natl. Acad. Sci. USA.

[16] Arrieta O., Aviles-Salas A., Orozco-Morales M., Hernández-Pedro N., Cardona A.F., Cabrera-Miranda L., Barrios-Bernal P., Soca-Chafre G., Cruz-Rico G., de Lourdes Peña-Torres M. (2020). Association between CD47 Expression, Clinical Characteristics and Prognosis in Patients with Advanced Non-small Cell Lung Cancer. Cancer Med..

[17] Cherciu I., Bărbălan A., Pirici D., Mărgăritescu C., Săftoiu A. (2014). Stem Cells, Colorectal Cancer and Cancer Stem Cell Markers Correlations. Curr. Health Sci. J..

[18] Eppert K., Takenaka K., Lechman E.R., Waldron L., Nilsson B., van Galen P., Metzeler K.H., Poeppl A., Ling V., Beyene J. (2011). Stem Cell Gene Expression Programs Influence Clinical Outcome in Human Leukemia. Nat. Med..

[19] Cho Y.-H., Ro E.J., Yoon J.-S., Mizutani T., Kang D.-W., Park J.-C., Il Kim T., Clevers H., Choi K.-Y. (2020). 5-FU Promotes Stemness of Colorectal Cancer via P53-Mediated WNT/β-Catenin Pathway Activation. Nat. Commun..

[20] Wang L.F., Choi H.S., Su Y., Lee B., Song J.J., Jang Y.-S., Seo J.-W. (2021). 7S,15R-Dihydroxy-16S,17S-Epoxy-Docosapentaenoic Acid, a Novel DHA Epoxy Derivative, Inhibits Colorectal Cancer Stemness through Repolarization of Tumor-Associated Macrophage Functions and the ROS/STAT3 Signaling Pathway. Antioxidants.

[21] Wang K., Song K., Ma Z., Yao Y., Liu C., Yang J., Xiao H., Zhang J., Zhang Y., Zhao W. (2020). Identification of EMT-Related High-Risk Stage II Colorectal Cancer and Characterisation of Metastasis-Related Genes. Br. J. Cancer.

[22] Yang Y., Ma L., Xu Y., Liu Y., Li W., Cai J., Zhang Y. (2020). Enalapril Overcomes Chemoresistance and Potentiates Antitumor Efficacy of 5-FU in Colorectal Cancer by Suppressing Proliferation, Angiogenesis, and NF-ΚB/STAT3-Regulated Proteins. Cell Death Dis..

[23] Liu H., Ren G., Wang T., Chen Y., Gong C., Bai Y., Wang B., Qi H., Shen J., Zhu L. (2015). Aberrantly Expressed Fra-1 by IL-6/STAT3 Transactivation Promotes Colorectal Cancer Aggressiveness through Epithelial–Mesenchymal Transition. Carcinogenesis.

[24] Oldenborg P.-A., Zheleznyak A., Fang Y.-F., Lagenaur C.F., Gresham H.D., Lindberg F.P. (2000). Role of CD47 as a Marker of Self on Red Blood Cells. Science.

[25] der Jeught K.V., Xu H.-C., Li Y.-J., Lu X.-B., Ji G. (2018). Drug Resistance and New Therapies in Colorectal Cancer. World J. Gastroenterol..

[26] Belgiovine C., D’Incalci M., Allavena P., Frapolli R. (2016). Tumor-Associated Macrophages and Anti-Tumor Therapies: Complex Links. Cell. Mol. Life Sci..

[27] Shmelkov S.V., Butler J.M., Hooper A.T., Hormigo A., Kushner J., Milde T., St. Clair R., Baljevic M., White I., Jin D.K. (2008). CD133 Expression Is Not Restricted to Stem Cells, and Both CD133+ and CD133– Metastatic Colon Cancer Cells Initiate Tumors. J. Clin. Invest..

[28] Fan Z., Duan J., Wang L., Xiao S., Li L., Yan X., Yao W., Wu L., Zhang S., Zhang Y. (2019). PTK2 Promotes Cancer Stem Cell Traits in Hepatocellular Carcinoma by Activating Wnt/β-Catenin Signaling. Cancer Lett..

[29] Luo F., Li J.B., Liu J.H., Liu K.P. (2022). Stabilizing and Upregulating Axin with Tankyrase Inhibitor Reverses 5-Fluorouracil Chemoresistance and Proliferation by Targeting the WNT/Caveolin-1 Axis in Colorectal Cancer Cells. Cancer Gene Ther..

[30] Johnson D.E., O’Keefe R.A., Grandis J.R. (2018). Targeting the IL-6/JAK/STAT3 Signalling Axis in Cancer. Nat. Rev. Clin. Oncol..

[31] Zhang T., Liu L., Lai W., Zeng Y., Xu H., Lan Q., Su P., Chu Z. (2019). Interaction with Tumor-associated Macrophages Promotes PRL-3-induced Invasion of Colorectal Cancer Cells via MAPK Pathway-induced EMT and NF-κB Signaling-induced Angiogenesis. Oncol. Rep..

[32] Schiavoni G., Gabriele L., Mattei F. (2013). The Tumor Microenvironment: A Pitch for Multiple Players. Front. Oncol..

[33] Buhrmann C., Kraehe P., Lueders C., Shayan P., Goel A., Shakibaei M. (2014). Curcumin Suppresses Crosstalk between Colon Cancer Stem Cells and Stromal Fibroblasts in the Tumor Microenvironment: Potential Role of EMT. PLoS ONE.

[34] Wang D., Feng F., Ma Y. (2022). Tumor-Associated Macrophages as Treatment Target in Colorectal Cancer. Front. Med. Sci. Res..

[35] Pikarsky E., Porat R.M., Stein I., Abramovitch R., Amit S., Kasem S., Gutkovich-Pyest E., Urieli-Shoval S., Galun E., Ben-Neriah Y. (2004). NF-ΚB Functions as a Tumour Promoter in Inflammation-Associated Cancer. Nature.

[36] Wang B.-D., Kline C.L.B., Pastor D.M., Olson T.L., Frank B., Luu T., Sharma A.K., Robertson G., Weirauch M.T., Patierno S.R. (2010). Prostate Apoptosis Response Protein 4 Sensitizes Human Colon Cancer Cells to Chemotherapeutic 5-FU through Mediation of an NFκB and MicroRNA Network. Mol. Cancer.

[37] Fu J., Xu Y., Yang Y., Liu Y., Ma L.L., Zhang Y.Y. (2019). Aspirin Suppresses Chemoresistance and Enhances Antitumor Activity of 5-Fu in 5-Fu-Resistant Colorectal Cancer by Abolishing 5-Fu-Induced NF-κB Activation. Sci. Rep..

[38] Shakibaei M., Kraehe P., Popper B., Shayan P., Goel A. (2015). Curcumin Potentiates Antitumor Activity of 5-Fluorouracil in a 3D Alginate Tumor Microenvironment of Colorectal Cancer. BMC Cancer.

[39] Ji M., Li W., He G., Zhu D., Lv S., Tang W., Jian M., Zheng P., Yang L., Qi Z. (2019). Zinc-A2-Glycoprotein 1 Promotes EMT in Colorectal Cancer by Filamin A Mediated Focal Adhesion Pathway. J. Cancer.

[40] Wei C., Yang C., Wang S., Shi D., Zhang C., Lin X., Xiong B. (2019). M2 Macrophages Confer Resistance to 5-Fluorouracil in Colorectal Cancer through the Activation of CCL22/PI3K/AKT Signaling. OncoTargets Ther..

[41] Lu Y., Zhang R.Z., Zhang X.J., Zhang B., Yao Q.H. (2020). Curcumin May Reverse 5-Fluorouracil Resistance on Colonic Cancer Cells by Regulating TET1-NKD-Wnt Signal Pathway to Inhibit the EMT Progress. Biomed. Pharmacother..

[42] Koga N., Hu Q., Sakai A., Takada K., Nakanishi R., Hisamatsu Y., Ando K., Kimura Y., Oki E., Oda Y. (2021). Clinical Significance of Signal Regulatory Protein Alpha (SIRPα) Expression in Esophageal Squamous Cell Carcinoma. Cancer Sci..

